# Inflammation and the Heart: The Role of Traditional Factors

**DOI:** 10.1155/crp/4623305

**Published:** 2026-07-29

**Authors:** Mostafa Cheraghi, Arash Amin, Solmaz Jabarzare, Toomaj Sabooteh, Parisa Moradi, Mostafa Dehghani, Mehrnoosh Sedighi, Fatemeh Hatami, Ali Rafieian, Tara Farshidfar

**Affiliations:** ^1^ Cardiovascular Research Center, Shahid Rahimi Hospital, Lorestan University of Medical Sciences, Khorramabad, Iran, lums.ac.ir; ^2^ Clinic Lipid, Madani Hospital, Lorestan University of Medical Sciences, Khorramabad, Iran, lums.ac.ir; ^3^ Medplex Family Practice, 25 Woodstream Blvd #6, Woodbridge L4L 7Y8, Toronto, Canada; ^4^ School of Medicine, Shahid Rahimi Clinical Research Development Center, Lorestan University of Medical Sciences, Khorramabad, Iran, lums.ac.ir; ^5^ Student Research Center, Faculty of Medicine, Shahid Beheshti University of Medical Sciences, Tehran, Iran, sbmu.ac.ir

**Keywords:** atherosclerosis, heart, inflammation, interleukins

## Abstract

About 50% of cardiac patients do not have typical cardiac risk factors. Therefore, it is important to search for additional factors that may be responsible for heart disease. If these factors are identified, the test could help predict high‐risk cardiovascular patients. The studies about the etiology of coronary artery disease have led to the discovery of novel biomarkers. Recently, accumulating evidence has highlighted the critical role of both systemic and localized inflammatory markers accompanied by atherosclerosis and cardiac conditions. Particularly, higher levels of inflammatory biomarkers like CRP can lead to cardiovascular diseases. This article presents an analysis of the causes of heart disease, atherosclerosis, endothelial dysfunction, and plaque rupture, with a special focus on the role of inflammation as a contributing factor. Additionally, the article examines ways to reduce the inflammation associated with cardiovascular disease.

## 1. Introduction

Chronic inflammatory diseases represent the most significant cause of mortality globally. The World Health Organization identifies chronic diseases as the greatest threat to public health. In 2000, nearly 125 million Americans were living with a chronic disease, with 61 million (21%) affected by more than one. By 2014, more recent estimates showed that approximately 60% of Americans had at least one chronic disease, 42% had multiple chronic conditions, and 12% of adults had 5 or more chronic diseases. It is estimated that three out of every five individuals worldwide die from chronic inflammatory diseases (CIDs). One of the most significant special diseases resulting from chronic inflammation is the group of conditions known as CIDs, which includes cardiovascular diseases (CVDs), diabetes, arthritis, and joint diseases. In its 2017 update, the American Heart Association stated that about 800,000 annual mortalities were due to CVDs in the United States. Globally, CVD represents 31% of total mortality, and coronary heart disease is the leading cause of CVD‐related mortality. In the United States, following CHD, stroke (responsible for 1 in 20 deaths) and heart failure represent significant contributors to cardiovascular mortality [1–4].

The evolution of inflammation as a defensive strategy has enabled the host organism to mount an effective response to potential threats. If the inflammation is not properly resolved, the acute inflammatory phase can progress to a chronic inflammatory state. The chronic inflammation happens when an underlying stimulus, like infection or chronic cellular damage, is not effectively removed [5]. It has been shown that inflammatory mediators such as TNF‐α, some interleukins, reactive oxygen species (ROS), and other types could affect the heart and vessels, influencing the metabolic processes of cardiomyocytes and endothelial cells [6]. However, increased levels of inflammatory markers are also linked to modifiable risk factors such as physical inactivity, dietary habits, obesity, and smoking [7].

A substantial corpus of studies indicates cardiomyopathy to be a medical condition characterized by the inflammation of cardiac tissues and with significant negative effects on heart health and function. Chronic inflammation can be the underlying factor in the development of CVDs, with the potential to result in severe complications, including heart failure and myocardial infarction (MI). Based on the evidence presented, there is a relationship between abnormal inflammatory processes and a wide range of CVDs. In recent decades, various studies have shown that the immune system and inflammation play a crucial role in the pathophysiology of CVDs, and the sustained expression of some inflammatory factors may cause various adverse effects on the heart [7–11]. Although the role of inflammatory factors and their associated risk factors in cardiovascular disorders has been extensively reviewed, the exact mechanisms by which these factors contribute to CVDs remain unclear. Since early diagnosis and treatment of CVDs are critical, this review aims to provide an updated and comprehensive overview of the identification of anti‐inflammatory markers and environmental factors and their effects on the cardiovascular system. Additionally, it presents the role of inflammatory biomarkers in enhancing risk assessment in high‐risk populations.

## 2. Materials and Methods

To conduct this review, relevant articles published between 1989 and 2024 were collected from scientific databases, Google Scholar, PubMed, Web of Science using the terms inflammation, atherosclerosis, heart, interleukins, interferons, chemokines, and traditional factors that interfere with heart function. Eligible articles were then reviewed.

## 3. Findings

### 3.1. The Key Inflammatory Mediators

There are several conditions that can lead to inflammatory responses, like viral, bacterial, autoimmune, and lifestyle‐related causes. Therefore, early diagnosis and appropriate treatment are the key points to control possible complications. It is important to focus on the prevention and treatment of heart inflammation in order to promote heart health [12]. The process of inflammation is initiated by the activation of specific pathogen receptors, which in turn will lead to the production of key inflammatory mediators. These mediators cause inflammation by modulating vascular endothelial permeability and recruiting neutrophils and excess plasma at the site of injury [13].

Immune cells target and eliminate invading pathogens. The duration of an inflammatory response is contingent upon the level of injury sustained. An excessive amount of these cytokines leads to an exaggerated response. The liver has a fundamental role in inflammation. In response to the triggers, the liver cells will produce and secrete inflammatory mediators such as C‐reactive protein (CRP) and various coagulation factors. Such effects are not merely localized; rather, they have a systemic impact [14]. Recent studies indicate that an inflammatory disorder that affects endothelial cells of the coronary arteries can lead to atherosclerosis. Additionally, instability and rupture of atherosclerotic plaques are due to inflammation, both at local and systemic levels, which in turn leads to acute cardiovascular events. The objective is to ascertain whether biomarkers of inflammation may facilitate the enhancement of risk assessment and the identification of at‐risk groups. One of the most utilized markers of the inflammatory process is CRP. In both its role as a causal factor and in its capacity as a predictor of CHD, it is the most extensively studied [15, 16]. CRP is a widely studied inflammatory marker, extensively researched in numerous global studies. Elevated CRP concentration is mainly associated with cardiac complications such as heart failure and cardiac death that usually take place following acute infarction [17]. Evidence suggests that CRP may contribute to the development of atherosclerotic lesions. This process leads to reduced expression of nitric oxide (NO) synthase and prostacyclin synthase. Additionally, it increases LDL‐C levels and promotes its uptake by macrophages, a key step in the progression of atherosclerosis. Moreover, CRP influences the expression of adhesion molecules in endothelial cells [18].

In the context of heart disease, additional inflammatory mediators, such as activated T cells and mast cells, have been observed to bind to the endothelium. The activation of macrophages, T lymphocytes, and smooth muscle cells results in the release of additional mediators. The collective actions of these inflammatory cells ultimately result in the formation of lipid reservoirs contained in atheromatous lesions, which are protected by a fibrous cap [19].

The synthesis of CRP is modulated by interleukin‐6. Moreover, IL‐6 levels are regulated with other cytokines [20]. It can be posited that CRP enhances the uptake of LDL by macrophages and augments their capacity to form foam cells. CRP is a potent procoagulant that can precipitate intravascular coagulation, leading to thrombosis during inflammatory conditions. CRP has been demonstrated to enhance the expression and activity of PAI‐1 (protease inhibitor), a major regulator of fibrinolysis that inhibits tissue plasminogen activator. Hence, elevated PAI‐1 would lead to atherosclerosis due to inhibition of fibrinolytic activity [21].

### 3.2. Atherosclerosis, Inflammation, and the Heart

Atherosclerosis is a chronic disease with low‐grade inflammatory conditions [22]. Both systemic and local inflammation can lead to endothelial dysfunction and progress toward symptomatic CVDs [23]. Inflammation, independent of traditional risk factors [24, 25], is predictive of CVD. Acute and chronic conditions, such as psychological stress, autoimmune diseases, infections, and aging, can provoke endothelial dysfunction [26]. This, in turn, amplifies a low‐grade inflammatory response with vascular involvement, driving the progression of heart disease [27]. This process not only contributes to coronary artery disease (CAD) but also has a key role in large vessel thrombotic stroke and cerebral aneurysms [28].

All stages of vascular disease, such as atherosclerosis, from the accumulation of atherogenic lipoproteins to plaque formation and rupture, have a complex network that involves the primary and secondary immune systems, in addition to the spleen and bone marrow [25]. Numerous studies have demonstrated that a significant portion of the beneficial effects of statins arises from their ability to decrease inflammatory responses, not only due to their lipid‐lowering actions [29]. In addition, nearly half of the patients receiving high‐intensity statin therapy for lipid reduction in secondary prevention trials, in addition to achieving significant lipid‐lowering effects, had a low‐grade inflammatory risk and lower major cardiovascular events [30]. Recent clinical trials have also shown that inflammation leads to a reduced incidence of CAD and stroke [31].

### 3.3. Oxidative Stress and Inflammation

Pathological inflammation is a multifaceted, cell‐wide process that initiates with the generation of excess free radicals, typically as a response to internal or environmental stressors originating from mitochondria. This initiates several signaling stages that ultimately produce substances responsible for the hallmark symptoms of inflammation [32]. Oxidative stress and, subsequently, B NF‐kappa lead to the increased production of inflammatory mediators. CD40‐CD40L interactions can trigger several signaling pathways through the immune and cardiovascular systems and modulate oxidative stress [33]. Excess ROS/RNS is not only responsible for inflammation [34], but it is also a primary driver of many human diseases [35]. These include dyslipidemia [36], thrombosis [37], metabolic syndrome [38], type 2 diabetes [39], non‐alcoholic steatohepatitis (NASH) [40], macular degeneration [41], and neurodegenerative diseases like Alzheimer’s [42]. Inflammation caused by oxidative stress has an important role in the development of CAD and thrombosis [43]. Oxidative stress and antioxidants also play a role in the pathogenesis and management of diabetes and diabetic cardiomyopathy [44]. Inflammatory conditions such as arthritis [45], congestive cardiomyopathy [46], and hypertension [47]. In addition, plasma vitamin E levels had an inverse relationship with mortality from ischemic heart disease [48]. In this regard, medicinal plants with antioxidant activities have been shown to reduce atherosclerosis [49].

### 3.4. CRP, Inflammation, and the Heart

Researchers are working to identify inflammatory markers that could improve the prediction of MI and stroke [50]. These studies focus on the potential role of inflammatory markers, including cytokines and intercellular adhesion molecules, on the progression of atherosclerosis and future cardiovascular events [51]. For instance, researchers discovered that the blood level of CRP is several hundred times higher in acute injury, infection, and inflammation. Antioxidants have been shown to reduce CRP [52]. Recent developments in high‐sensitivity measurements for CRP (hs‐CRP) have led to the understanding that CRP serves as a marker of microinflammation. It has been identified that even healthy people with higher levels of CRP are at increased risk of future cardiovascular events [53]. Hs‐CRP measurements are the most powerful indicator of cardiovascular risk compared to the rest of the inflammatory markers. Patients with severe CAD exhibited the highest levels of hs‐CRP [54].

### 3.5. IL‐1β, Inflammation, and Heart

Inflammation activates the proinflammatory factor pro‐IL‐1β, turning it into its active form, IL‐1β. This, in fact, strengthens the inflammatory response by promoting the development of atherosclerotic plaque and inducing some structural changes that lead to plaque instability and rupture. Additionally, interleukin 1β can induce the blood level of interleukin 6 and lead to increased CRP production in liver cells [55]. Canakinumab is a human monoclonal antibody targeting IL‐1β. It will inhibit IL‐1β from attaching to its receptor without affecting IL‐1α [56]. 150 mg of canakinumab every 3 months will reduce 15% MACE compared to the placebo [57]. This effect mainly reduced the incidence of MI; however, the mortality rate remained the same. The significant reduction in hs‐CRP with no change in LDL‐C has led to a reduced rate of MI cases, resulting from the role of IL‐1β being blocked from interacting with its receptors. This shows that targeting inflammation through the inhibition of the IL‐1β pathway is important for managing CRP levels. Despite its known effect of CANTOS in the IL‐1β pathway modulation, the FDA did not approve using canakinumab for ASCVD patients due to a notable rise in lethal infections and sepsis [58].

### 3.6. TNF‐α, Inflammation, and Heart

Tumor necrosis factor alpha is one of the strongest proinflammatory cytokines. Thereby, TNF‐α inhibitors are widely used for the management of systemic inflammatory diseases. About 1 million patients are receiving the treatment with TNF‐α antagonists, making this drug class the most lucrative globally, generating annual sales of $25 billion [59]. TNF‐α is a signaling protein widely distributed throughout the body, primarily synthesized by macrophages. Additionally, various other cell types, including cardiac myocytes, can produce TNF‐α as well. The TNF‐α molecule is present in two distinct forms: one bound to the membrane and the other found in the cytosol [60]. The primary role of TNF‐α is to regulate the immune system by interacting with its specific cell membrane receptors, TNFR1 and TNFR2 [61]. Its effects are wide‐ranging, including physiological influences on heart health as well as pathological contributions to the development of heart diseases [62]. In a mouse model of MI, TNF‐α is toxic through TNFR1 activation, but protective due to TNFR2 activation [63]. This duality may help explain the conflicting results observed in various studies, where some reported detrimental effects [61], while others highlighted a protective role [64]. The TNF‐α inhibitors are primarily used in the treatment of autoimmune inflammatory diseases, but they could be a potential treatment for patients with heart systolic failure and diabetes. These patients typically exhibit high blood levels of anti‐TNF‐α and other cytokines [65]. The better heart function observed in these patients is linked to reduced levels of TNF‐α [66]. Heart failure patients often exhibit increased cardiac TNF‐α levels, which are associated with changes in the expression of TNFRI and TNFR2 [67]. Additionally, genetic studies have shown that polymorphisms in the TNFA gene are linked to an elevated risk of CAD [68] and relate to a higher risk of gastrointestinal complications in CAD [69]. Studies showed that anti‐TNF‐α treatment in individuals with autoimmune inflammatory diseases for a long period of time may provide protection against an increase in cardiovascular complications and cardiovascular mortality [70]. Although some cases of heart failure with reduced ejection fraction have been reported in these patients [71], this chance is low in patients under 50 who are receiving anti‐TNF‐α drugs like etanercept or infliximab [72]. Anti‐TNF‐α medications are mainly used for managing rheumatoid arthritis, and they showed better improvement in their left ventricular diastolic function [73]. In fact, RA patients who are treated with infliximab have less damage to their left ventricle [74]. Additionally, anti‐TNF‐α therapy can protect against vascular diseases, including atherosclerosis. Rheumatoid arthritis patients who received anti‐TNF‐α treatment showed lower levels of soluble endothelial adhesion molecules [75] and improved arterial stiffness [76] and endothelial function [77]. The use of TNF‐α inhibitors can reduce the risk of MI [78] and acute coronary syndrome [79], highlighting anti‐TNF‐α therapy as an effective treatment in RA patients [80].

### 3.7. IL‐6, Inflammation, and Heart

IL‐6 has two types: membrane‐bound gp130 receptor and soluble IL‐6R. When there is injury to the tissue or infection, IL‐6 will stimulate the immune system, which will contribute to the acute immune response and fever. IL‐6 was initially considered as a cytokine derived from T cells, although it is now recognized that it is synthesized by a variety of cell types and exerts its broad biological effects through binding to a distinctive receptor complex [78]. Additionally, there is a correlation between IL‐6 levels and ischemic heart diseases and acute MI [81]. Notably, elevated IL‐6 levels were strongly linked to heart function in AMI patients, a correlation that was not observed in chronic heart failure [82]. Furthermore, IL‐6 plasma level was higher in MI patients with cardiogenic shock compared to uncomplicated acute MI cases, which means IL‐6 might be correlated with MI complications [83]. A clinical trial study showed patients with elevated levels of IL‐6 had poorer outcomes following acute coronary syndrome [84]. Moreover, in patients with stable CAD, IL‐6 level was correlated to a higher chance of cardiovascular‐related complications, like heart failure [85]. Several experimental studies emphasized the relation between IL‐6 level and ischemia/reperfusion outcomes. These studies showed IL‐6 plasma level was noticeably higher 4 h following left coronary artery obstruction and 2 h following reperfusion in rabbits [86]. Additionally, mRNA levels of IL‐6 and gp130 were increased in the hearts of mice after MI [87]. These studies indicate that IL‐6 level is related to the infarct size in the early stage of reperfusion. For example, lower levels of IL‐6 were accompanied by a smaller size of infarct following 1 h of ischemia and 3 h of reperfusion in mice [88]. Furthermore, an elevation in sIL‐6R levels has been observed in patients with AMI, indicating that targeting IL‐6R inhibition may offer a promising therapeutic approach for managing MI [89]. A recent study by Hartman and colleagues in mice demonstrated that although using monoclonal antibodies MR16‐1 in order to block IL‐6R could not stop the heart remodeling following I/R, a single dose of the IL‐6R antagonist, such as tocilizumab, somehow inhibited the release of troponin T, which is responsible for the inflammatory response in NSTEMI patients [90]. Additionally, VCAM‐1 level increased during the hospital stay of patients who had received tocilizumab without affecting their coronary flow [91].

### 3.8. IL‐1, Inflammation, and Heart

The interleukin‐1 (IL‐1) is a family of 11 proinflammatory cytokines, which are all involved in triggering and managing inflammatory reactions that can cause acute and chronic diseases. Among these 11 proinflammatory cytokines, interleukin 1α and interleukin 1β are studied the most. The researchers confirmed the presence of a naturally designated antagonist called IL‐1Ra that can regulate both interleukin 1α and interleukin 1β by attaching to the interleukin 1 receptor [92]. The interleukin 1R antagonist is regarded as an anti‐inflammatory cytokine [93].

Interleukin 1 exerts a negative inotropic effect in two different settings: firstly, in isolated cardiomyocytes, and secondly, in intact hearts [94]. The role of IL‐1 has been confirmed in different cardiovascular conditions such as MI, cardiomyopathy, and heart failure [95]. An increased level of IL‐1, especially IL‐1β, was found in acute MI patients. AMI is the predisposing factor of cardiac I/R injury that is accompanied by a high level of IL‐1 cytokines, notably IL‐1β, which will help in the establishment of I/R [96]. Conversely, lowering the amount of IL‐1β through protective modalities can help to reduce cardiac I/R injury. Experimental studies on mice or rats showed that using IL‐1 receptor antagonists has a protective effect on the myocardium and could control the size of cardiac infarcts [97]. Moreover, the suppression of inflammatory cytokine production has been demonstrated to contribute to the maintenance of cardiac function in donor hearts following transplantation [98]. These findings suggest a potential therapeutic value of the IL‐1Ra gene in preserving the myocardium [99]. Mauro and colleagues [100] have recently demonstrated that the inhibition of IL‐1α has a protective effect against cardiac I/R injury, reduces the size of infarction, and can preserve LV function through inhibiting the inflammatory response in mice. Consequently, the inhibition of interleukin‐1α could lead to a significant protective approach for mitigating I/R‐induced cardiac injury. In this regard, consumption of flaxseed extract has been associated with a reduction in inflammatory activation and decreased levels of interleukins [101]. In conclusion, the interleukin‐1 level is related to myocardial injury and can lead to myocarditis or dilated cardiomyopathy. As a result, it is considered that lowering the level of IL‐1 can have a protective effect on myocarditis, acute MI, and IHD. Moreover, utilizing IL‐1 receptor inhibitors as a therapeutic approach for treating various CVDs presents a promising direction for future research [102].

### 3.9. IL‐8, Inflammation, and Heart

The process of atherosclerosis starts with the attachment of peripheral blood monocytes to the artery wall. When the vascular wall is damaged, it will express various kinds of cytokines that will attract leukocytes, thereby facilitating their adhesion to the endothelium and allowing them to migrate trans‐endothelial into the subendothelial space [103]. IL‐8 and MCP‐1 have been shown to be stimulated by various agents, including elevated glucose levels, oxidized low‐density lipoprotein, homocysteine, and NO. Interleukin 8 can be excreted from subendothelial macrophages [104]. The presence of IL‐8 within existing atherosclerotic plaques can lead to an increase in matrix metalloproteinases (MMPs) that are derived from foam cells originating from macrophages. Additionally, interleukin 8 can modulate the production of oxLDL in a dose‐dependent manner. Patients who are diagnosed with unstable CAD have been found to display elevated levels of IL‐8 in their plasma in comparison to those diagnosed with stable CAD and the control group. There is a relation between interleukin 8 and WBC level; studies have also revealed that there are high levels of IL‐8 and white cells in patients with CAD. The observations presented here suggest that the potential of IL‐8 to impact the risk of CAD is in a manner independent of white blood cell attraction, likely through a destabilizing effect on atherosclerotic plaques resulting from a decrease in tissue inhibitors of metalloproteinases (TIMPs). The existing body of prospective data supports the concept that IL‐8 is involved in the progression of symptomatic CAD [105, 106].

### 3.10. IL‐18, Inflammation, and Heart

A notable observation revealed in acute MI and the subsequent myocardial damage is that there is a high level of interleukin‐18. However, elevated plasma IL‐18 levels have been reported not to be associated with myocardial necrosis or the level of the creatine kinase enzyme [107]. Interleukin 18 is defined as an inducer for IFN‐γ. As such, IFN‐γ has been demonstrated to increase the expression of both chemokines and adhesion molecules in inflammatory responses [108]. The expression of adhesion molecules to the endothelium happens in two ways: first, in an IFN‐γ‐dependent manner, and second, in an IFN‐γ‐independent manner via IL‐18 [109]. The pharmaceutical compound under investigation has been demonstrated to have tumor necrosis factor alpha (TNF‐α), interleukin 1, and interleukin 6 associated with an increased risk of cardiovascular disorders [110]. Consequently, serum concentrations of IL‐18 in IHD patients without known cardiovascular risk factors were higher in comparison to healthy individuals. Thereby, interleukin‐18 can be considered an independent risk factor for ischemic heart diseases [111].

### 3.11. IL‐17, Inflammation, and Heart

The findings of the studies have demonstrated that the serum level of interleukin 17 was high in patients with acute MI and urinary tract infection. Interleukin 17 is produced by a group of T cells called T helper 17. Additionally, interleukin 23 would trigger the production of interleukin 17 [112]. Consequently, the role of interleukin 17 and 23 in CVDs needs to be studied further. Evidence showed that there is a high level of T helper 17 in patients with acute coronary syndrome [113]. The present findings suggest that IL‐17 exerts its principal effect as a proinflammatory mediator by means of various mechanisms, including, but not limited to, the stimulation of the production of other proinflammatory cytokines. It also exerts a stimulatory effect on the production of certain chemokines, including, notably, XCL1 and XCL2. This process can result in the formation of adhesion molecules [114–116]. It is evident that IL‐17 also modulates the attraction of neutrophils and monocytes to the site of inflammation. This process is induced by the expression of various chemotactic mediators, such as IL‐8, MCP‐1, and growth‐related protein (Gro)‐α. The present findings suggest that an elevated level of interleukin 17 can be an indicator for ischemic heart disease progression [115, 117].

### 3.12. IL‐10, Inflammation, and Heart

In the aftermath of a MI, a substantial influx of leukocytes is observed in necrosis, with the primary function of these cells being the removal of necrotic debris. As part of the body’s innate stress response to myocardial damage, there is an increase in proinflammatory cytokines within the first hour of the incident [118]. In the Days 3–5 following the heart attack, the initial inflammatory response transition starts with the production and activation of fibroblasts [119]. Inflammation constitutes a pivotal element in the initial phases of left ventricular remodeling. However, prolonged inflammation can lead to left ventricular dilatation, overproduction of scar tissue in LV walls, and, subsequently, impaired LV function [120]. Thus, it has been demonstrated that time is a vital factor in inhibiting inflammatory responses. In the Days 1–7 following a heart attack, repair macrophages are activated; they have a critical role in the inhibition of inflammatory reactions by releasing suppressive factors like TGF‐β and IL‐10 [121]. IL‐10 has been demonstrated to possess a robust capacity to suppress pre‐inflammation [122]. In animal models, significant increases in serum IL‐10 levels have been observed within 6 h of myocardial ischemia/reperfusion [123]. IL‐10 is demonstrated to inhibit the release of proinflammatory mediators in different cell types [124]. In patients suffering from acute MI, elevated serum IL‐10 levels within 24 h of undergoing angioplasty were associated with a lower rate of heart failure exacerbation [125]. The studies conducted on mice revealed that a lower level of IL‐10 was accompanied by a larger infarct size and myocardial necrosis due to higher infiltration of neutrophils [126]. In the aftermath of MI, the administration of IL‐10 therapy has been demonstrated to attenuate inflammatory responses and enhance LV function [127].

### 3.13. TGF‐β, Inflammation, and Heart

Inflammatory response following MI can generate healing and scar tissue in the affected myocardium [120, 128]. The process of myocardial repair and regeneration may be subdivided into three stages: the inflammatory, proliferation, and maturation phases. It has been established that during the inflammatory phase, there is a concomitant production of free radicals, the initiation of the complement cascade, and the activation of signaling pathways mediated by nuclear factor (NF)‐kB and toll‐like receptors (TLRs). These phenomena result in heart death and hypoxia. Because of these occurrences, the synthesis of chemokines and cytokines is initiated. The synthesis of these compounds then results in the regulation of the adhesion molecules and leukocytes, which will lead to the infiltration of monocytes, lymphocytes, and polymorphonuclear cells into the infarct area. The initial phase of wound healing is the removal of dead cells and matrix debris by neutrophils and macrophages. After this initial phase, the inflammatory mediators would be suppressed, and fibroblast and endothelial cells would invade the wound. As most inflammatory cells are susceptible to apoptosis, activated myofibroblasts will secrete extracellular matrix, resulting in the development of a substantial microvascular network, which will form scar tissue. The cellular and molecular events that occur in the aftermath of a MI have a direct impact on the remodeling of the left ventricle. These events predict the prognosis of patients who have been hospitalized with cardiovascular events. TGF‐β modulates numerous processes associated with infarct healing. It has been hypothesized that TGF‐β would recruit monocytes in the healing phase after a MI and that it may contribute to the formation of granulation tissue and suppression of inflammatory responses following MI. Additionally, TGF‐β regulates fibrous tissue deposition through the mediation of a myofibroblastic phenotype, the induction of extracellular matrix protein synthesis, and the promotion of matrix preservation by increasing TIMPs. It has been demonstrated that femtomolar concentrations of transforming growth factor‐β1 (TGF‐β1) can induce a chemotactic response in both neutrophils and monocytes [129]. Studies showed that TGF‐β1 has an inhibitory effect on endothelial tissue and can decrease leukocyte adhesion and neutrophil migration in the endothelial layer due to reduced expression of E‐selectin on endothelial surfaces [130]. Moreover, TGF‐β has a suppressive effect on macrophages by inhibiting peripheral monocytes from converting to macrophages. Thereby, TGF‐β1 will suppress the synthesis of proinflammatory cytokines and chemokines [131, 132].

### 3.14. IL‐2, Inflammation, and Heart

Interleukin 2 was first identified as a growth factor for T lymphocytes originating from the bone marrow of an organism. Interleukin 2 has pleiotropic effects on various cell types. These effects include the activation of T cell growth and the augmentation of the activity of natural killer cells. Studies suggest that interleukin 2 enhances the transformation of regulatory T cells and increases immunoglobulin production by B cells and plays a role in apoptosis. Moreover, IL‐2 is essential for the body’s immune response to microbial infections and helps protect against autoimmune diseases. As demonstrated in studies, IL‐2 exerts its effects through the process of binding to IL‐2 receptors [133]. A body of research exists that suggests a correlation between IL‐2 and numerous cardiovascular conditions. It was evident that cancerous patients who received high doses of interleukin 2 had a higher rate of acute MI and myocarditis [134]. In this specific context, it is proposed that serial echocardiography (an analysis of the pattern of transmittal Doppler flow) might serve as a predictive indicator of cardiovascular complications, with the aim of facilitating the prevention of complications arising from high‐dose IL‐2 administration [135].

Although in patients suffering from acute or stable ischemic events, there are higher levels of interleukin 2, studies have demonstrated that interleukin 2 has potential therapeutic effects in acute MI. It has been documented that 50 U/mL of IL‐2 exhibits a comparable effect to that of ischemic preconditioning, as indicated by a reduction in infarct size and LDH release. This phenomenon transpires through a process that involves the stimulation of Kappa‐opioid receptors [136].

### 3.15. IL‐4, Inflammation, and Heart

Research has demonstrated that the administration of a long‐acting interleukin 4 enhances cardiac outcome in a murine model of acute MI. The subsequent administration of interleukin 4 following the onset of MI has been shown to increase the population of macrophages. As demonstrated by the expression of a range of anti‐inflammatory and tissue repair‐related genes in the damaged myocardium. Consequently, this resulted in enhanced cardiac repair and diminished adverse myocardial remodeling. This is characterized by the formation of locally dense and thick fibrous tissues in the walls of the infarcted ventricle, as well as augmented microvascular formation in the myocardium [137, 138]. Additionally, the systolic and diastolic ventricular function exhibited marked enhancement with a significantly reduced rate of cardiac dilation. This study suggested that administration of IL‐4 in the aftermath of MI has the potential to augment the intrinsic repair response by activating M2 macrophages. Therefore, interleukin 4 has a potential therapeutic effect in acute MI [139, 140].

It has been established that M2 macrophages extracted from the heart following MI exhibited high levels of profibrotic factors, including TGF‐β and osteopontin [141]. As indicated by reports, potential side effects of interleukin 4 include pathological fibrosis and arteriosclerosis. These findings have given rise to concerns regarding its clinical application [142].

### 3.16. Exercise, Inflammation, and Heart

Regular exercise has been demonstrated to modulate the proinflammatory response in two pathways: interleukin 6‐dependent and interleukin 6‐independent. Doing regular exercises can help in the elevated production of adiponectin, which has anti‐inflammatory and insulin‐sensitizing effects [143]. Furthermore, regular exercise can help in reducing weight and is linked to decreased levels of hs‐CRP. Furthermore, consistent exercise has a positive impact on lipid profiles and helps in managing blood pressure. All these effects will help in controlling inflammation. Studies showed that doing regular exercise is an independent indicator that can control inflammatory responses, and it is not related to the other inflammatory indicators. The experimental studies on mice revealed that 6 h following the exercise, the blood level of administered bacterial lipopolysaccharides was lower compared to the group of mice that did not exercise [144].

The extensive body of evidence suggests that doing regular exercise will reduce proinflammatory cytokine production in muscular tissues. Additionally, the amount of cytokine production from monocytes was lower. The positive effects of exercise on endothelial function have been demonstrated, including reduced activation of endothelial cells and, as a result, fewer adhesion molecules and chemokine expression [145].

This study aims to enhance the production of anti‐inflammatory cytokines while simultaneously suppressing inflammatory cytokines. A 6‐month period was selected as the basis for the study, and research findings demonstrated a correlation between increased exercise levels and elevated HDL cholesterol levels. These results indicated a significant increase in HDL cholesterol levels from the baseline (pre‐exercise regimen) over time, which is related to reduced CVDs [146, 147]. A plethora of pharmacological interventions have been demonstrated to augment plasma HDL levels. This has been correlated with a diminution in the risk of major cardiovascular incidents [148].

In CAD, a comprehensive training regimen spanning 4 weeks has been shown to result in a significant enhancement of the vasodilator response to acetylcholine, a key neurotransmitter. This phenomenon has been linked to an augmentation in the expression of total endothelial NO synthase, in addition to protein kinase B (Akt) phosphorylation. The stimulatory effect of exercise on NO production has been corroborated by findings from animal studies [149]. Furthermore, a study revealed that the aorta of sedentary mice displayed heightened levels of lipid peroxidation and vascular superoxide compared to the aorta of mice that had been subjected to voluntary running for a period of 6 weeks [150].

### 3.17. Smoking, Inflammation, and the Heart

Evidence has demonstrated that smoking cessation leads to reduced risk of atherosclerotic cardiovascular disease (ASCVD) through a variety of mechanisms. This includes a significant decrease in inflammatory processes. A negative correlation between the number of years elapsed since smoking cessation and levels of hs‐CRP, as well as white blood cells, total cholesterol, triglycerides, and systolic blood pressure, was observed [151–153].

A two‐factorial randomized controlled trial was conducted on sedentary women. An investigative study was carried out. In this study, smokers were assigned at random to a smoking cessation program that incorporated exercise. It has been documented that inflammatory markers in accordance with white blood cells were reduced [154]. In a study encompassing 784 participants who smoked, the implementation of bupropion for smoking cessation purposes was found to be linked to a statistically significant decrease in hs‐CRP levels 7 weeks after the preliminary baseline assessment. The continuation of bupropion treatment in a patient with low white blood cells and neutrophil levels is indicated, given the significant benefits demonstrated in the treatment of ASCVD. The product has been associated with smoking cessation and is recommended for individuals who smoke. The cessation of smoking has been proven to be an effective method to reduce the risk of atherosclerotic CVDs [155]. The utilization of FDA‐approved cessation medications has been shown to facilitate this process [156].

### 3.18. Omega‐3, Fatty Acids, Inflammation, and Heart

In the REDUCE‐IT trial, evidence suggested that statin therapy can improve cardiac function regardless of the baseline serum triglyceride levels. The samples were randomly divided into highly pure eicosatetraenoic acid (EPA). The 4 g/day formulation of EPA led to 25% ischemic events and mortality compared to the placebo [157]. A statistically significant reduction in levels of hs‐CRP was observed among those who were randomized to receive icosapent ethyl as compared to those who received a placebo. The mean percent change from baseline was 12.6% versus 29.9% (*p* < 0.001), indicating a potential anti‐inflammatory effect with EPA supplementation. A reduction in triglyceride levels has been demonstrated to mediate some of the potential anti‐inflammatory effects of EPA. In addition, the EPA serves as an antecedent to a series of specialized lipid mediators known as “resolvins,” which have been demonstrated to inhibit inflammatory processes and facilitate the resolution of chronic inflammation [158]. Evidence has demonstrated that omega‐3 fatty acids possess the ability to reduce cytokine release and decrease the activation of endothelial and platelet cells, which may partially elucidate their observed anti‐inflammatory effects. Subsequent studies are presently being conducted with the aim of elucidating the precise mechanisms through which the anti‐inflammatory effects of EPA are manifested [159].

### 3.19. Obesity, Inflammation, and Heart

Insulin‐resistant adipose tissue displays metabolic activity. Furthermore, a variety of inflammatory mediators are released, which contribute to the production of monocyte chemoattractant protein‐1. Adipocytes can trigger inflammation by recruiting inflammatory cells into their interstitial space [160]. The findings from the PET scan suggested the existence of inflammation in the visceral adipose tissue, notably in omental and hepatic fat. Research showed that subcutaneous fat does not carry the same amount of local inflammation. Liposuction surgery in obese patients with significant abdominal volume did not lead to a substantial decrease in inflammatory markers. Conversely, patients with obesity who undergo bariatric surgery and subsequently experience weight loss have demonstrated a substantial reduction in inflammatory biomarker concentrations [161]. An increase in visceral obesity has been demonstrated to coincide with notable elevations in blood inflammatory biomarkers. These findings could be due to both insulin and leptin resistance [162]. In addition, several inflammatory pathways have been identified as mediating the association between obesity and metabolic syndrome, as well as the elevated risk of atherosclerosis. For instance, TNF‐α, a proinflammatory cytokine, is secreted by macrophages that infiltrate adipose tissue. Furthermore, release of fatty acids from adipose tissue in accordance with advanced glycation can lead to inflammatory and oxidative reactions [160].

### 3.20. Diet, Inflammation, and the Heart

A substantial body of observational research has indicated that there is a relation between unhealthy eating and inflammation. In a randomized crossover study design, a diet rich in saturated fat has been associated with elevated serum levels of CRP and E‐selectin in healthy adult males [163]. In type 2 diabetic patients, the consumption of a diet with high saturated fat content has been associated with increased levels of various inflammatory markers [164]. A growing body of research has established a link between the consumption of dietary trans‐hydrogenated fats and an elevated risk of vascular inflammation. This phenomenon is primarily driven by the induction of NF‐κB and IL‐6 production [165].

In addition, a high intake of trans fats increased the level of CRP and fibrinogen [166] and the risk of ischemic heart diseases [167]. Conversely, a diet or a plant abundant in polyunsaturated fatty acids has been linked to a decrease in inflammatory cytokines [168]. A body of research in the epidemiological field has indicated a correlation between a diet with high fiber content and a reduced risk of CVDs. As indicated by the relevant research, there is a positive correlation between elevated sugar intake and increased plasma levels of the inflammatory markers [169]. Moreover, this process is concomitant with augmented NF‐κB activation within mononuclear cells, as previously documented [170].

### 3.21. Age, Inflammation, and Heart

A robust body of literature has identified a direct correlation between the aging process and the development of both CVD and systemic inflammation. It is evident that this process ultimately results in the development of atherosclerosis, a condition characterized by the formation of plaques in blood vessels. Bone marrow hematopoietic stem cells (HSCs) are responsible for the production of all blood cell types, including immune cells [171]. Bone marrow vessels are similarly subject to stimulation by other tissues. Evidence has demonstrated a relationship between inflammation and the function of bone marrow arterioles. Consequently, this process leads to the increased proliferation of HSCs. Moreover, studies have demonstrated a rise in the release of leukocytes into the bloodstream with advancing age [172].

Subsequently, the release of cytokines and proteases by these leukocytes occurs after their absorption by activated endothelial cells. Furthermore, the cells migrate to the arterial wall, where they contribute to an increase in inflammation [173]. A body of research in the epidemiological field has demonstrated a correlation between the process of aging and the occurrence of somatic mutations in HSCs. These mutations have been shown to have the potential to lead to the development of leukemia [174]. The gradual buildup of somatic mutations can lead to the expansion of a mutant leukocyte clone, progressively altering its immunological properties [175]. This establishes a novel correlation between the process of aging and the development of atherosclerosis [176]. Furthermore, as individuals age, the sympathetic nervous system’s activation in response to psychological stressors leads to an elevation in the concentration of noradrenaline in the bone marrow; this process is known to promote the proliferation of HSCs, with a particular emphasis on myeloid cells. The result of this proliferation is an increased release of leukocytes into the circulation [177].

### 3.22. Endothelial Function, Inflammation, and Heart

The vascular endothelium has been demonstrated to exert a substantial influence on the development of CVDs. In healthy individuals, the endothelium, a monolayer of cells that lines the interior surfaces of blood vessels, exerts anti‐inflammatory and antithrombotic effects, thereby regulating the permeability of circulating molecules. Additionally, vascular tone is modulated by the balance between the secretion of vasodilators, such as NO, and the release of constrictors derived from the endothelium, including endothelin [178]. Several factors, including bacterial and viral infections and environmental factors, contribute to the condition. Stress has been shown to reduce the levels of NO in the body. The phenomenon under investigation has been shown to result in damage to endothelial connections and an increase in permeability to macromolecules [179].

A substantial body of research has evidenced a robust correlation between low‐density lipoprotein and the development of atherosclerosis [179, 180]. Upon entering the subendothelial space, low‐density lipoprotein undergoes oxidation and forms large complexes through aggregation. Additionally, in an inflammatory environment, the process of lipoprotein metabolism is altered. The transformation of large and medium‐sized low‐density lipoprotein particles into small and dense subfractions is a critical process in the context of cardiovascular health and disease. This compound exhibits a reduced affinity for the liver‐specific low‐density lipoprotein receptor [181]. Elevated levels of small and dense subfractions of low‐density lipoprotein have been associated with an increased risk of developing CAD [182].

**FIGURE 1:**
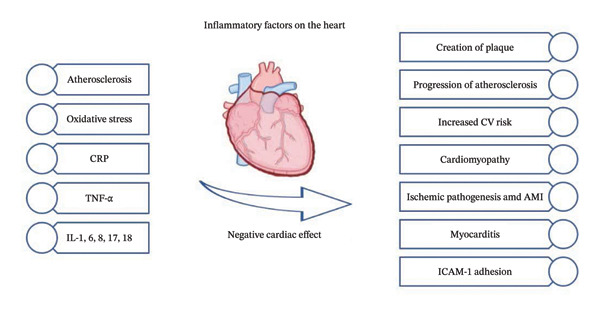
The role of oxidative stress and inflammatory factors on the heart. CRP: C‐reactive protein; TNF‐α: tumor necrosis factor; IL‐1‐18: interleukin 1‐18; CV: cardiovascular disease; AMI: asterisk manager interface; ICAM‐1: intercellular adhesion molecule.

**FIGURE 2:**
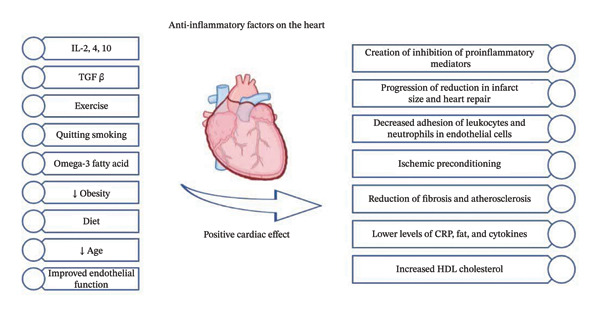
Anti‐inflammatory factors effective on the heart. IL: interleukin; TGF‐β: transforming growth factor beta; CRP: C‐reactive protein; HDL: high‐density lipoprotein.

The exposure of the arterial wall to sdLDL‐C, along with its atherogenic activity and oxidation, is a key factor in the progression of this condition. Additionally, in recent years, there has been a notable rise in type 2 diabetes and obesity, coupled with the advent of effective therapeutic interventions for LDL‐C control. The lipid risk profile of the population has undergone a notable shift, with an increase in triglyceride‐rich lipoproteins (TRLs) and a concomitant reduction in low‐density lipoprotein cholesterol. Moreover, residual lipoproteins (RLPs) have been identified as a significant contributor to the inflammatory response, with a higher potency than that observed in LDL‐C [183, 184]. The effects of inflammatory and anti‐inflammatory factors on the heart are illustrated in Figures 1 and 2, respectively.

### 3.23. Signaling Events Involved in Inflammation

**FIGURE 3:**
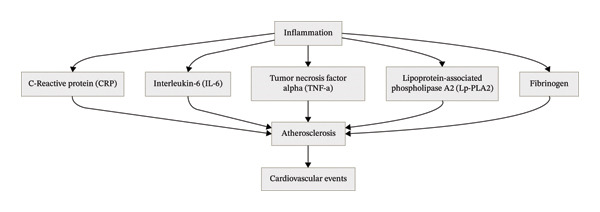
Inflammatory biomarkers involved in cardiovascular disease.

To facilitate comprehension among readers regarding the impact of inflammation on heart disease, a comprehensive overview of the signaling events implicated in this process was provided, which is illustrated in Figure 3. A major cause of raised levels of several indicators, including CRP, interleukin‐6 (IL‐6), TNF‐α, lipoprotein‐associated phospholipase A2 (Lp‐PLA2), and fibrinogen, is inflammation, which is linked to the onset of atherosclerosis, a major cause of cardiovascular events, including strokes and heart attacks (Figure 3).

### 3.24. Pharmacological Therapeutic Approaches to CVDs

The understanding of inflammation as a key component in the development of atherosclerosis and CVD has led to advancements in medical therapies aimed at inflammatory pathways. The current treatment regimens for CVD continue to prioritize the management of conventional risk factors, such as hypertension, dyslipidemia, and diabetes, with the use of antiplatelet therapy, including statins, beta‐blockers, angiotensin‐converting enzyme inhibitors, and other pharmacological agents. However, emerging data suggest that anti‐inflammatory therapies can be used to reduce cardiovascular incidents in people at high risk. Statins have proven to be essential since they reduce blood lipid levels and have secondary anti‐inflammatory effects. These secondary effects include the reduction of circulating levels of CRP, which has been identified as a predictor of cardiovascular risk. Recent studies have revealed reductions in cardiovascular events unrelated to lipid‐lowering with novel medications targeting inflammatory pathways, such as interleukin‐1β inhibitors. This discovery supports the theory that inflammation is the root cause of cardiovascular illnesses. In the context of heart failure and other inflammation‐related cardiovascular illnesses, sodium–glucose cotransporter 2 inhibitors (SGLT2i) have been shown to reduce cardiovascular death and hospitalizations, likely via a number of mechanisms, such as anti‐inflammatory effects. To improve adherence and treat numerous cardiovascular risk pathways at once, including inflammation, fixed‐dose combination therapy is being studied. The ongoing study of pharmacological modulation of endothelial dysfunction, plaque stabilization, and the immune response holds significant potential for expanding treatment options. Including anti‐inflammatory drugs is one example of this. The integration of pharmacotherapy into the cardiovascular treatment approach is a significant step forward in addressing residual cardiovascular risk, particularly in patients who have elevated inflammatory biomarker profiles but do not exhibit typical risk factors. New pharmacological agents to decrease inflammation and minimize cardiovascular risk include the use of colchicine and canakinumab.

Changes in diet, exercise, and other lifestyle variables can help lower inflammation and improve cardiovascular health [185].

### 3.25. Suggestions for Future Work

1. Future studies should test whether adding markers like hs‐CRP, interleukin‐6, TNF‐α, and other cytokines improves the ability to discriminate those at risk. Particular attention should be paid to validating these enhanced models in different populations (by age, sex, and ethnicity) and determining their incremental benefit over existing tools.
2. Large, well‐designed randomized controlled trials assessing both efficacy (reducing cardiovascular events) and safety over long follow‐up periods are recommended.
3. A lot of plants have been introduced with anti‐inflammatory and antioxidant potential (188). Studies combining the extracts of these plants with standard therapies (e.g., statins, antihypertensives) to see if there is an additive or synergistic benefit are recommended.
4. Mapping how inflammatory signaling differs in acute versus chronic cardiac injury (e.g., post‐MI vs. chronic heart failure) is also important.

## 4. Conclusion

Inflammation is a significant contributing factor in CVDs. Preliminary studies indicate that inflammation is closely associated with cardiovascular outcomes in healthy and high‐risk individuals. The inflammatory mediators are also linked to other risk elements of acute coronary syndrome. The advent of highly sensitive assays, such as CRP, has the potential to enhance the accuracy of cardiovascular risk prediction and expedite the initiation of preventive or secondary treatments for atherosclerosis and other CVDs.

## Author Contributions

Conceptualization: Mehrnoosh Sedighi, Fatemeh Hatami, Ali Rafieian, and Tara Farshidfar. Methodology: Arash Amin, Mehrnoosh Sedighi, and Mostafa Cheraghi. Writing–original draft preparation: Fatemeh Hatami and Ali Rafieian. Writing–review and editing: Mostafa Cheraghi, Arash Amin, Solmaz Jabarzare, Toomaj Sabooteh, Parisa Moradi, Mostafa Dehghani, Mostafa Dehghani, Fatemeh Hatami, and Ali Rafieian.

## Funding

This study was supported financially by Cardiovascular Research Center of Lorestan University of Medical Sciences, Khorramabad, Iran.

## Disclosure

All authors agreed to be accountable for the research presented.

## Ethics Statement

The authors have nothing to report.

## Conflicts of Interest

The authors declare no conflicts of interest.

## Data Availability

Data sharing is not applicable to this article as no new data were created or analyzed in this study.
